# Pimobendan Inhibits HBV Transcription and Replication by Suppressing HBV Promoters Activity

**DOI:** 10.3389/fphar.2022.837115

**Published:** 2022-06-03

**Authors:** Si-Yu Yuan, Hai-Bo Yu, Zhen Yang, Yi-Ping Qin, Ji-Hua Ren, Sheng-Tao Cheng, Fang Ren, Betty Yuen Kwan Law, Vincent Kam Wai Wong, Jerome P. L. Ng, Yu-Jiao Zhou, Xin He, Ming Tan, Zhen-Zhen Zhang, Juan Chen

**Affiliations:** ^1^ The Key Laboratory of Molecular Biology of Infectious Diseases Designated by the Chinese Ministry of Education, Chongqing Medical University, Chongqing, China; ^2^ State Key Laboratory of Quality Research in Chinese Medicine, Macau University of Science and Technology, Macao, China; ^3^ Chongqing Key Laboratory of Child Infection and Immunity, Ministry of Education Key Laboratory of Child Development and Disorders, National Clinical Research Center for Child Health and Disorders, Department of Infectious Diseases, The Children’s Hospital of Chongqing Medical University, Chongqing, China

**Keywords:** hepatitis B virus, HBsAg, pimobendan, anti-HBV agents, drug repurposing

## Abstract

Current anti-HBV therapeutic strategy relies on interferon and nucleos(t)ide-type drugs with the limitation of functional cure, inducing hepatitis B surface antigen (HBsAg) loss in very few patients. Notably, the level of HBsAg has been established as an accurate indicator to evaluate the drug efficacy and predict the disease prognosis, thus exploring a novel drug targeting HBsAg will be of great significance. Herein, by screening 978 compounds from an FDA-approved drug library and determining the inhibitory function of each drug on HBsAg level in HepG2.2.15 cells supernatant, we identified that pimobendan (Pim) has a powerful antiviral activity with relatively low cytotoxicity. The inhibitory effect of Pim on HBsAg as well as other HBV markers was validated in HBV-infected cell models and HBV-transgenic mice. Mechanistically, real-time PCR and dual-luciferase reporter assay were applied to identify the partial correlation of transcription factor CAAT enhancer-binding protein α (C/EBPα) with the cccDNA transcription regulated by Pim. This indicates Pim is an inhibitor of HBV transcription through suppressing HBV promoters to reduce HBV RNAs levels and HBsAg production. In conclusion, Pim was identified to be a transcription inhibitor of cccDNA, thereby inhibiting HBsAg and other HBV replicative intermediates both *in vitro* and *in vivo*. This report may provide a promising lead for the development of new anti-HBV agent.

## Introduction

Hepatitis B virus infection remains a global public health problem, about 257 million people are living with chronic HBV infection (CHB), which is a leading cause of liver cirrhosis and hepatocellular carcinoma (Trépo et al., 2014; Lanini et al., 2019). HBV contains a 3.2-kb circular, partially double-stranded DNA genome that can be converted into episomal covalently closed circular DNA (cccDNA) inside the nucleus of infected hepatocytes. The cccDNA serves as a template for encoding four overlapping open reading frames that produce seven proteins, namely large, middle, and small surface proteins, HBV core protein, polymerase, hepatitis B e antigen, and hepatitis B x protein, and all of these proteins play an irreplaceable role in viral transcription and replication. Importantly, cccDNA has been identified to cause a long-term persistence and chronic infection, thus elimination of cccDNA becomes the key to curing HBV (Lucifora and Protzer, 2016). The currently available treatments for CHB include nucleoside analogs (NAs) such as Entecavir (ETV), Tenofovir and Lamivudine, which function as nucleoside reverse transcription inhibitors to suppress HBV replication. Immunotherapies such as pegylated interferons also play an important role in clinical treatment. Although both therapies can reduce viral load (HBV DNA), neither can completely eliminate virus cccDNA nor lead to serum HBsAg clearance. In addition, NAs are used as a lifelong treatment for CHB, leading to a high risk of drug resistance, and IFNs are poorly tolerated in patients. Thus, developing novel anti-viral strategies for CHB is highly desirable.

Several definitions of cure are proposed by the American Association for the Study of Liver Disease and the European Association for Study of the Liver (Kramvis, 2020). HBsAg, one of the oldest and best evaluated bio-markers for HBV infection in clinics, plays a critical role in diagnosis and treatment. The loss of HBsAg is defined as “functional cure”, and subsequently, the seroconversion with detectable anti-HBs antibody levels is considered as the ideal realistic treatment endpoint (Mueller et al., 2018; Kramvis, 2020). Studies also show that quantitative HBsAg levels not only have a correlation with cccDNA level, but also provide additional predictability of serological and clinical outcomes, thus being as good predictors of treatment response (Liu et al., 2016). HBsAg suppressive agents will provide alternative therapies for chronic HBV infection and reduce the risk of developing severe liver diseases.

The process for a new drug to be approved can take 10–15 years and is expensive. This long discovery process opens the doors for drug repurposing as an approach to cutting down the time required to develop a drug (Parvathaneni et al., 2019). Thus, in order to bring laboratory discovery into the clinical setting more efficiently, the US Food and Drug Administration (FDA)-approved drug library was chosen to conduct a phenotypic screening for inhibitors of secreted HBsAg, among which pimobendan (Pim) stood out from 978 compounds. Pim is a benzimidazole-pyridazinone derivative that is approved by the FDA for the management of the symptoms of mild, moderate, or severe congestive heart failure in dogs due to chronic valvular heart disease or dilated cardiomyopathy (Boyle and Leech, 2012). In Japan, Pim is clinically available for inotropic treatment in patients with advanced heart failure or as a supportive treatment for beta-blocker therapy (Murai et al., 2013; Nonaka et al., 2015). It has been reported that this compound works by a dual mechanism, calcium sensitizers and phosphodiesterase inhibitors. In particular, Pim inhibits phosphodiesterase to reduce the breakdown of cyclic adenosine monophosphate and exhibits a positive inotropic effect (Boyle and Leech, 2012; Guzman et al., 2014; Nonaka et al., 2015). Additionally, Pim exhibits several pharmacological effects, such as catecholamine secretion, antithrombotic, rhythm and insulinotropic effects (Boyle and Leech, 2012). However, the effect of Pim on chronic HBV infection is yet to discover. Herein, we firstly reported the anti-HBV activity of Pim and its underlying mechanism. We found that the Pim treatment decreased intracellular and supernatant HBsAg, as well as other HBV replicative intermediates. Importantly, we further confirmed the inhibition of HBV by Pim *in vivo*. Mechanistically, the inhibition of Pim on cccDNA transcription was related to the transcription factors, of which the transcription factor C/EBPα was mostly implicated. Taken together, our results suggest the potential use of Pim as an alternative therapeutic strategy for HBV treatment.

## Materials and Methods

### Compounds and Plasmids

The FDA-approved compounds library was purchased from Selleck (FDA-approved drug library, L1300). Pim was purchased from MedChemExpress (MCE, CAS Number: 74150-27–9). Pim was dissolved in DMSO and stored at −80°C with a concentration of 500 mM, and diluted with medium to desired concentrations upon use. Plasmids pGL3-Cp, pGL3-Xp, pGL3-SpⅠ and pGL3-SpⅡ were constructed through replacing the SV40 promoter with four HBV inner promoters into vector pGL3-basic. C/EBPα was constructed by in-frame insertion of full-length C/EBPα into pcDNA3.1.

### Cell Culture

HepG2.2.15 and HepAD38 cell lines were purchased from the Shanghai Second Military University, and cultured in Dulbecco’s Modified Eagle’s Medium (DMEM, Sigma) supplemented with 10% fetal bovine serum and 100U/ml penicillin and 0.1 mg/ml streptomycin (Hyclone, sv30010) in the presence of G418 (Merck). HepG2-NTCP cell was a gift from Ningshao Xia (Xia Men University, Xia Men, China) and 2 μg/ml of doxycycline was needed to induce the expression of the NTCP receptor. Huh-7 cells were obtained from Health Science Research Resource Bank, HepG2-NTCP and Huh-7 cells were maintained in DMEM supplemented with 10% fetal bovine serum and 100U/ml penicillin and 0.1 mg/ml streptomycin. Primary human hepatocytes (PHH) were acquired from ScienCell Research Laboratories, and maintained in a hepatocyte medium (5210, ScienCell, United States). All cells were cultured at 37°C in a 5% CO_2_ incubator.

### Virus Production and Infection

HBV inocula were prepared as previously described (Cheng et al., 2021). Briefly, HBV particles (genotype D) were collected from the supernatant of HepAD38 cells, concentrated with 5% polyethylene glycol 8000 precipitation. Following gently rotated overnight at 4°C, the mixture was centrifuged at 4,000×rpm for 30 min at 4°C to collect HBV particles, the pellet was resuspended in serum-free opti-MEM at 1% volume of the original supernatant samples. Virus stocks were aliquoted and stored at −80°C. HepG2-NTCP cells were seeded in collagen-coated plates, infected with 1 × 10^3^ gene equivalents/cell of HBV particles in the presence of 4% polyethylene glycol 8000 for 24 h, the cells were washed three times with phosphate buffered saline (PBS) for further experiments.

### MTT and the Alamar Blue Assay

The cytotoxic effects of Pim on various cells were assessed by MTT assay (Sangon Biotech). Cells were seeded in 96-well plates and then exposed to the tested compound at different concentrations or DMSO as a control for 72 h. Subsequently, 40 μg/ml MTT solution was added to each well and incubated for 4 h protected from light. The medium was then removed and 100 μL of DMSO was added into each well to dissolve the formazan formed in the cells. After shaking at low speed for 10 min at room temperature, the colorimetric reading of the solute mixture was determined by spectrophotometer (BioTek, VT Lab, United States) at OD 490 nm. The fifty-percent cytotoxicity concentrations (CC_50_) were calculated via non-linear regression using GraphPad Prism 8.0.

Alamar Blue assay (Invitrogen) was performed by following the manufacturer’s instructions. In brief, the medium was removed from 12-wells plate after treatment, then rinsed cells with PBS and added 1 ml of alamar blue solution. After 3 h incubation in dark, 100 μL of the medium was transferred to a 96-well plate, and fluorescence was detected at the respective excitation and emission wavelength of 560 and 590 nm using a microplate reader.

### RNA Extraction and Real-Time PCR

Cellular total RNA isolation and cDNA synthesis were performed using TRNzol reagent (DP424, TIANGEN, China) and FastKing RT kit (KR116-02, TIANGEN, China). Quantitative PCR experiments were carried out using Fast Start universal SYBR Green Master (1725122, Bio-Rad) according to the manufacturer’s instruction in QuantStudio six Flex (Thermo Fisher Scientific). The β-actin mRNA was used to normalize target gene experiments. The 2^-∆∆Ct^ method was applied to calculate the fold changes of the gene expressions of all RT-PCR reactions.

### HBV Core Particle Extraction and Quantitation

Cells were washed with PBS and lysed in lysis buffer (50 mM Tris-HCl pH 7.4, 1 mM EDTA, and 1% NP-40) at 37°C for 15 min, the nuclei were removed after centrifugation at 10,000 g for 1 min 10 μL cytoplasmic cell lysate was collected to detect β-actin by western blot. The remaining cytoplasmic cell lysate was incubated with 100 mg/ml DNase Ⅰ and 100 mM MgCl_2_ for 30 min at 37°C to digest free nucleic acids. The reaction was stopped by adding EDTA to a final concentration of 25 mM. Protein was digested with 0.5 mg/ml proteinase K and 1% SDS for 2 h at 50°C (Belloni et al., 2012), Following purified by phenol/chloroform (1:1), the HBV DNA was precipitated with ethanol and dissolved in TE buffer.

HBV core DNA was quantified by using SYBR Green Mix (Bio-Rad, 1725122) in the Bio-Rad real-time quantitative PCR detection system. The sequences of the primers were listed in the Supplementary Table.

### HBV Hirt DNA Extraction and Analysis

HBV cccDNA was extracted from HBV-infected cells by a modified Hirt method as previously described (Gao et al., 2019), HBV cccDNA levels were detected by Taq-man probe PCR. Before used as the templates for Taq-man probe PCR, the following treatment is necessary: 1) heating at 80 °C for 5 min and cooling on ice immediately to denature rcDNA; 2) incubating with exonuclease V (M0345S, New England Biolabs, MA, United States) at 37°C for 30 min to digest rcDNA; 3) heating at 100 °C for 20 min to denature cccDNA. The specific sequence of primers and probe were listed in Supplementary Table.

### Northern Blot

Total RNA was extracted by TRNzol reagent (DP424, TIANGEN, China) as described before, DIG Northern Starter Kit (12039672910, Roche, Mannheim, Germany) was used following the manufacturer’s instructions. The extracted total RNA was separated by formaldehyde-agarose gel, then transferring the RNA on nylon membrane by capillary siphon method and fixed by UV-cross linking. The membrane was hybridized with the DIG-labeled HBV RNA probe, after blocked and incubated with anti-digoxin secondary antibody at 37°C for 30 min, the signal of membrane was exposed by X-ray film.

### Southern Blot

The DIG-High Prime DNA Labeling and Detection Starter Kit (12039672910, Roche, Mannheim, Germany) was available in this experience, following the manufacturer’s instructions, the HBV core DNA or hirt DNA was separated by agarose gel, then transferred on nylon membrane by capillary siphon method and fixed by UV-cross linking. After hybridizing with a DIG-labeled full-length HBV genome probe, blocked and incubated with the anti-digoxin secondary antibody, the signal was detected by X-ray film.

### Western Blot and Dot Blot

Cells were lysed in a radioimmunoprecipitation assay lysis buffer with protease inhibitors. Protein concentration was detected by Pierce™ BCA Protein Assay Kit (23225, Thermo Fisher scientific), 30ug protein lysate was separated on SDS-PAGE and transferred to polyvinylidene fluoride membranes (10600023, GE Healthcare, UK). After blocking with 5% skim milk, membranes were incubated overnight at 4°C with the appropriate primary antibodies. The next day the membrane was incubated with the corresponding Horseradish Peroxidase -conjugated secondary antibody and the signals were visualized with ECL Western blot reagents (WBKLS0500, Millipore, Massachusettts, United States). GAPDH was used as a loading control. Rabbit anti-HBsAg polyclonal antibody (NB100-62652) was obtained from Novus (United States), mouse monoclonal antibody of C/EBPα(sc-365318), rabbit anti-β-actin monoclonal antibody (sc-47778) and mouse anti-GAPDH monoclonal antibody (sc-47724) were obtained from Santa Cruz Biotechnology (United States). Rabbit anti-PCNA monoclonal antibody (#13110) was obtained from Cell Signaling Technology. HBsAg dot blot assay was detected as described (Dougherty et al., 2007). The membrane was blocked and detected with the anti-HBsAg antibody (Novus, United States).

### Enzyme-Linked Immunosorbent Assay (ELISA)

Cells were treated with different concentrations of Pim, HBsAg in cell culture supernatant and mouse serum were detected using commercial enzyme-linked immunosorbent assay kits (KHB, China) according to the manufacturer’s instructions. The absorbance at 450 and 630 nm were measured.

### Dual-Luciferase Reporter Assay

Luciferase activity detection was measured by a dual-luciferase reporter assay system (Promega, United States) using the GloMax microplate luminometer (Promega, United States). The luciferase report plasmids (pGL3-Cp, pGL3-Xp, pGL3-SpⅠ and pGL3-SpⅡ) were transfected into HepG2-NTCP cells, the plasmid pRL-TK was cotransfected to normalize transfection efficiency. 12 h after transfection, the cells were treated with 15 or 30 μM Pim, and incubated for 48 h to detect the fluorescence intensity.

### IHC

Formalin-fixed and paraffin-embedded liver tissue from the mouse model were sectioned into slices, after deparaffinization and antigen retrieval, the sections were incubated with primary antibody (anti-HBs, working solution, ZM-0122, Zhongshan Jinqiao Biological Technology) overnight at 4°C, followed incubated with secondary antibody. Diaminobenzidine staining was used to determine immunoreactivity. Counterstaining nucleus were performed using hematoxylin.

### Animal Experience

HBV-transgenic mice (HBV-Tg C57 BL/6), encoding a 1.2-overlength copy of the HBV genome (serotype ayw), were kindly provided by Prof. Xia Ningshao (Xia Men University, China). The mice were housed and maintained under specific pathogen-free conditions. Mice were selected as age (6–8 weeks), weight (21 ± 1 g), and basically possess similar serum HBsAg and other markers. Then divided them into four groups: negative control (PBS), positive control (0.02 mg/kg ETV), low concentration test group (10 mg/kg Pim) and high concentration test group (30 mg/kg Pim). Animals receive all drugs via oral gavage every day. The serum samples were obtained via orbital blood every 4 days, all mice were sacrificed and the liver samples were isolated for intrahepatic HBV markers detection. All the animal experiments were carried out in accordance with the guidelines of the Chinese Council on Animal Care and approved by the Chongqing Medical University.

### Statistical Analysis

The significant differences between two groups were statistically analyzed by the non-parametric Mann-Whitney *U*-test. All statistical analyzes were performed by GraphPad Prism 8 (GraphPad Software Inc., San Diego, CA). Differences were considered significant when *p* < 0.05. Data are shown as mean value ±standard error from three independent experiments.

## Results

### Screening of FDA-Approved Compounds as Potential HBsAg Inhibitors

**FIGURE 1:**
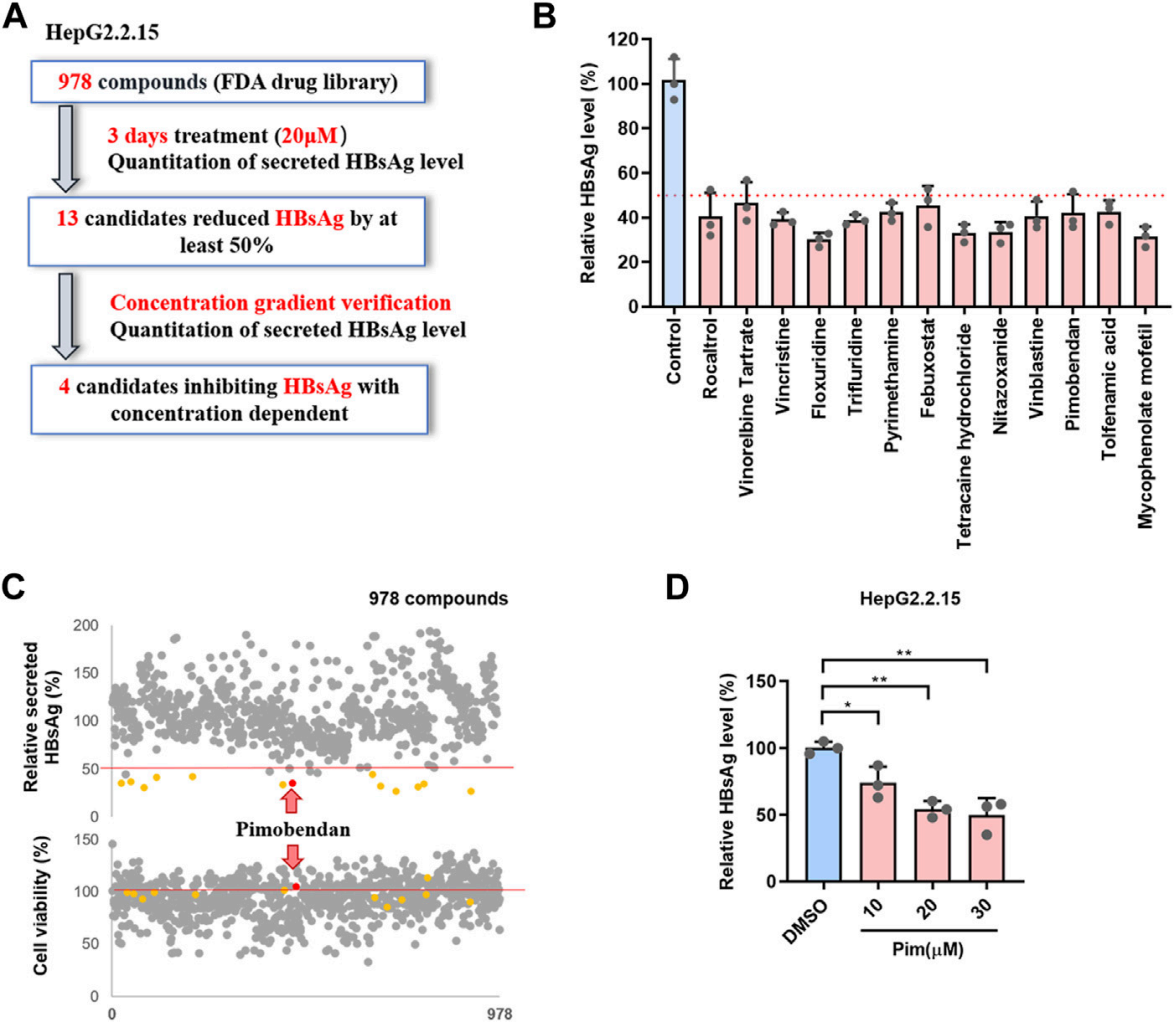
Screening of FDA-approved compounds as potential HBsAg inhibitors **(A)** Schematic depiction of a screen of FDA-approved compound library. HepG2.2.15 cells were treated with 978 FDA-approved drugs (20 μM) for 3 days, the secreted HBsAg level was quantitated **(B)** The 13 candidates suppressed HBsAg was tested by ELISA **(C)** The effects of all compounds on the supernatant HBsAg of HepG2.2.15 were detected by ELISA, and the effects on cell viability of HepG2.2.15 were detected by MTT **(D)** Pim inhibited the supernatant HBsAg in a dose dependent was detected by ELISA.

To discover potential HBsAg inhibitors, a phenotypic high-throughput screen of 978 compounds in an FDA-approved library was performed to detect the extracellular HBsAg level, and the detailed workflow was shown in Figure 1A. In the first step of screening, HepG2.2.15 cells, an HBV-replicating cell line carrying HBV DNA and stably secreting surface antigen particles (Li et al., 2012), were exposed to 20 μM of each compound for 3 days, and subsequently the inhibitory rate of compounds on HBsAg was detected by ELISA. It turned out that 13 candidates reduced HBsAg by at least 50% (Figure 1B and Supplementary Table S1). For the second confirmation screening, those 13 candidates were further assessed for inhibiting HBsAg production in a dose-dependent manner within the specified concentration range (Supplementary Figure S1). The cytotoxicity of those compounds on HepG2.2.15 cells was examined by MTT assay (Figure 1C). Among those selected compounds, Pim showed the best inhibition on secreted HBsAg of HBV in a dose dependent manner (Figure 1D), and exhibited relatively low cytotoxicity. Hence, Pim was considered as a promising compound for further investigations on its anti-HBV effects.

### Cytotoxicity and Anti-HBV Activity of Pim

**FIGURE 2:**
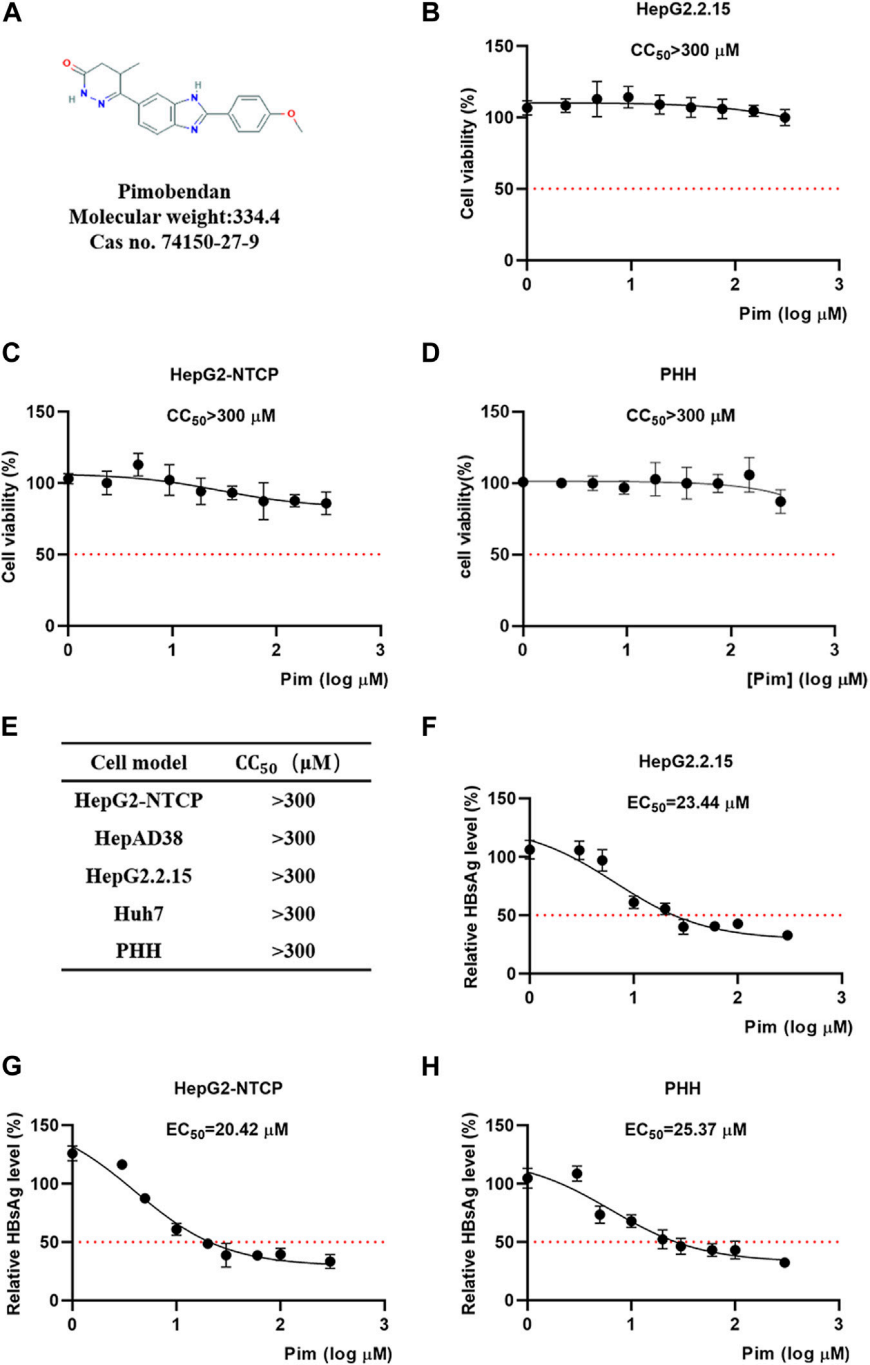
Cytotoxicity and anti-HBV activity of Pim **(A)** The chemical structure of Pim **(B–D)** Effects of Pim on the cell viability of HepG2.2.15, HepG2-NTCP and PHH cells were determined by the MTT assay **(E)** Indicated cell lines were treated with various concentrations of Pim for 72 h and its 50% cytotoxic concentration was determined **(F–H)** The EC_50_ of Pim on the inhibition of HBsAg in HepG2.2.15, HepG2-NTCP and PHH were determined by the ELISA assay. Pim treatment was started 72 h post HBV infection, and further treatment of Pim for 6 days in HepG2-NTCP and PHH cells.

The chemical structure of Pim was shown in Figure 2A. The cytotoxicity of Pim was examined by MTT conversion assay, which was conducted in various HepG2 hepatoma-derived cell lines including HepG2.2.15 (Figure 2B), HepG2-NTCP (Figure 2C) and HepAD38, primary human hepatocytes (Figure 2D) and HCC cell line Huh-7 (Supplementary Figure S2). Pim was found to exhibit no significant cytotoxicity against those cell lines with CC_50_ > 300 μM after treatment from 0 to 300 μM. All data were tabulated in Figure 2E. Further experiments revealed that the EC_50_ of Pim on inhibition of HBsAg was approximately 23.44 μM in HepG2.2.15 (Figure 2F), 20.42 μM in HepG2-NTCP (Figure 2G) and 25.37 μM in PHH (Figure 2H). Importantly, all selectivity indexes were >12.

### Pim Inhibits HBsAg Production and HBV Replication

**FIGURE 3:**
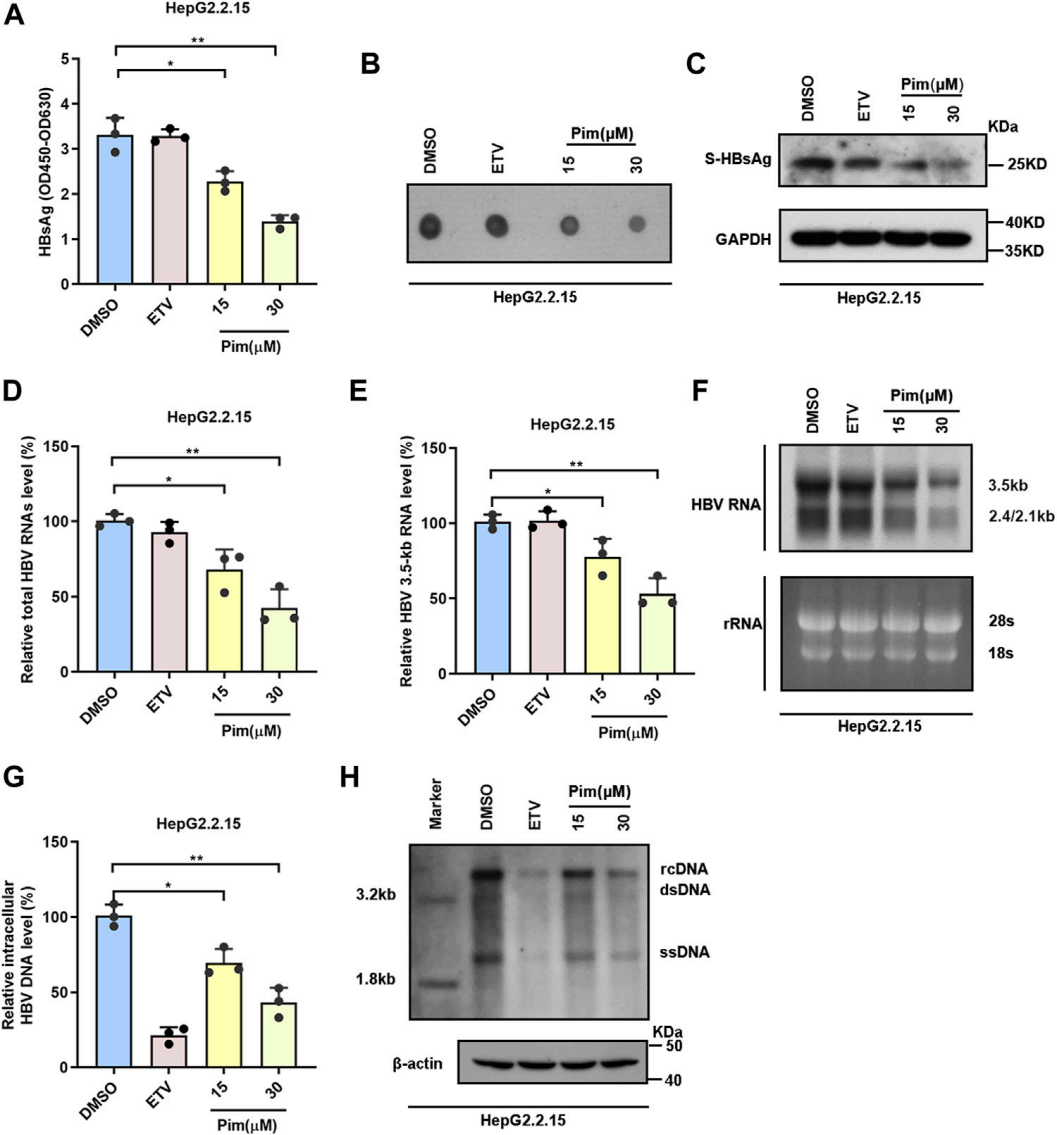
Pim inhibits HBsAg production and HBV replication **(A–B)** HepG2.2.15 cells were treated with DMSO, 25 nM ETV and 15 or 30 μM Pim respectively. the HBsAg level in the supernatant were determined by ELISA and dot blot assay **(C)** Western blot was used to detect the intracellular HBsAg in HepG2.2.15 **(D–E)** In HepG2.2.15 cells, relative real-time PCR was subjected to detect the total HBV RNAs and HBV 3.5-kb RNA levels, The mRNA level of β-actin was used as internal control **(F)** Northern blot was applied to determine the HBV RNAs, the rRNA level of 28S and 18S were used as an internal control **(G–H)** The quantification PCR and Southern blot were performed to determine the level of HBV core DNA after Pim treatment in HepG2.2.15, The level of β-actin was used as a loading control for HBV core DNA. The data are the mean ± SD from three independent experiments. rcDNA, relaxed circular DNA; dsDNA, double-strand DNA; ssDNA, single-strand DNA (*p < 0.05; **p < 0.01).

To elucidate the effects of Pim in inhibiting HBV, besides the secreted HBsAg, other HBV markers including intracellular HBsAg, HBV RNAs and HBV core DNA in HepG2.2.15 were assessed; HepG2.2.15 cells were treated with DMSO, 25 nM ETV and 15 μM or 30 μM Pim respectively. The inhibitory effect of Pim on HBsAg was first evaluated by ELISA assay and dot blot (Figure 3A,B). The results showed that the HBsAg level in cell supernatant was reduced by Pim treatment in a dose dependent manner. Western blot analysis also illustrated a decrease in intracellular HBsAg level upon dose dependent treatment with Pim (Figure 3C). It implied that Pim may affect the production of HBsAg. As HBV proteins were translated from HBV RNAs, we further analyzed the effect of Pim on HBV RNAs in HepG2.2.15. The real-time PCR results demonstrated that both the total HBV RNAs and HBV 3.5-kb RNA were decreased by Pim also in a dose dependent manner (Figure 3D,E). Consistently, results from the northern blot showed that 3.5-kb RNA and 2.4/2.1-kb RNA levels were suppressed by Pim (Figure 3F). Owing to HBV DNA replication intermediates originating from reverse transcription of HBV pregenomic RNA, the HBV core DNA was further examined by quantitative real-time PCR (Figure 3G) and southern blot (Figure 3H). Given the suppression in HBV pregenomic RNA level, the significant reduction of HBV core DNA was also verified. Taken together, these results suggested that Pim effectively inhibited HBsAg production and HBV replication in HepG2.2.15 cells.

### Anti-HBV Effects of Pim in HBV Infection Cell Model

**FIGURE 4:**
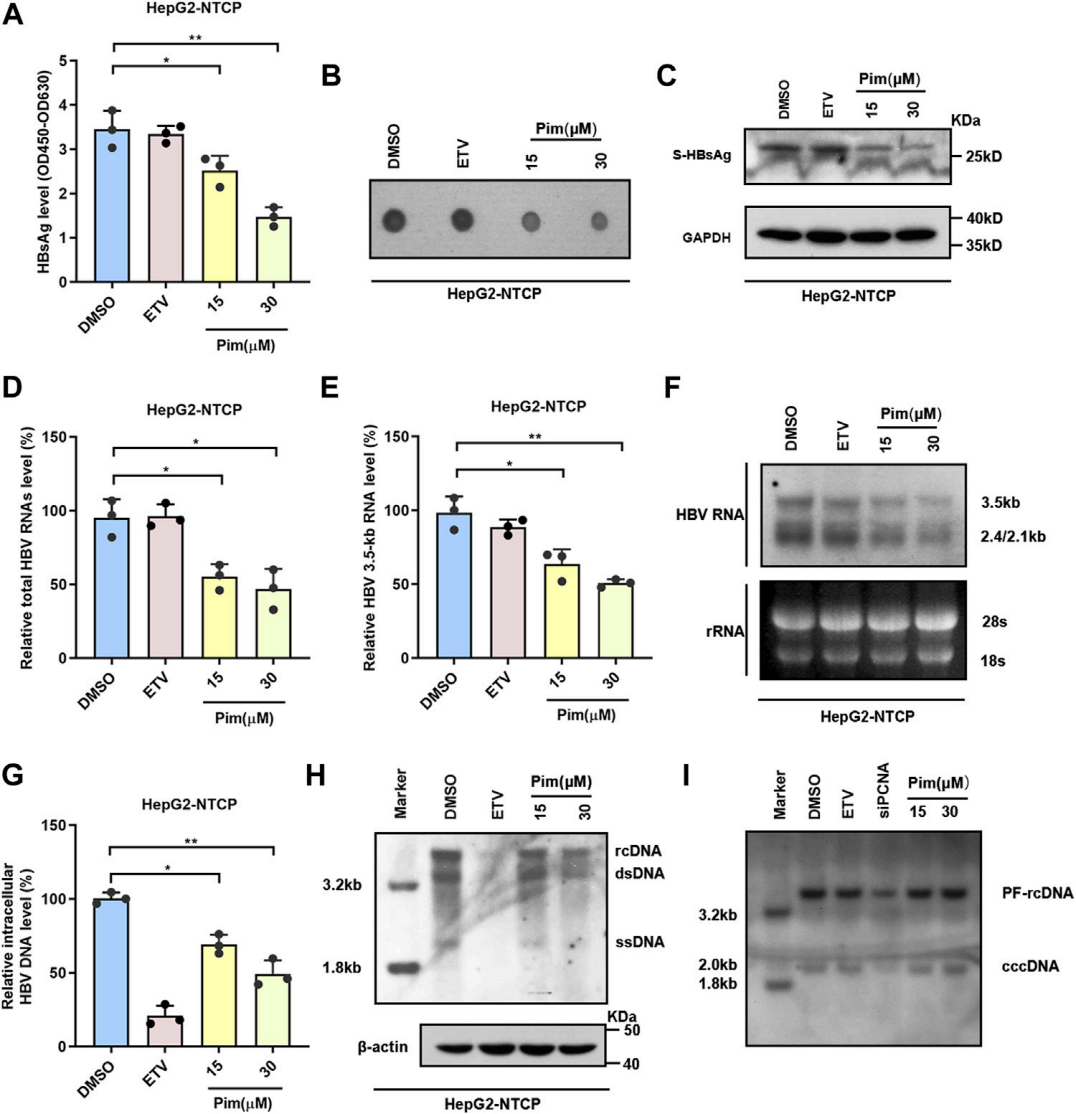
Anti-HBV effects of Pim in HBV infection model **(A)** HepG2-NTCP was treated with 15 or 30 μM Pim, 25 nM ETV as the positive control, and DMSO is negative control. Pim treatment was started 72 h post HBV infection, and further treatment of Pim for 6 days. ELISA assay was applied to determine the secreted HBsAg **(B)** The supernatant HBsAg level in HepG2-NTCP was determined by dot blot **(C)** Intracellular expression of HBsAg was confirmed by western blot in HBV-infected HepG2-NTCP **(D–E)** The total HBV RNAs and HBV 3.5-kb RNA were detected by real-time PCR after Pim treatment **(F)** HBV RNAs were analyzed by Northern blotting hybridization. Ribosomal RNAs (28S and 18S) served as loading control **(G–H)** The HBV core DNA in Pim treated HepG2-NTCP cells was observed by the quantitative PCR and southern blotting, respectively. The level of β-actin was used as a loading control for HBV core DNA. rcDNA, relaxed circular DNA. dsDNA, double-strand DNA. ssDNA, single-strand DNA **(I)** The cccDNA level after Pim treatment detected by Southern blot, siPCNA was used as a positive control for decreasing cccDNA (*p < 0.05; **p < 0.01).

It is well known that the main obstacle to curing chronic HBV infection is the persistence of cccDNA, and therefore the inhibitory effect of Pim on HBsAg produced from cccDNA was examined. HepG2-NTCP, a commonly used infection cell model, was employed to study the characteristics of HBV and validate the anti-HBV activity of Pim (Yan et al., 2012; Konig et al., 2019). As depicted in the experimental flowchart, Pim treatment started 72 h post HBV infection, and further treatment of Pim for 6 days (Supplementary Figure S3A). HBV infected HepG2-NTCP cells were treated with DMSO, 25 nM ETV and 15 μM or 30 μM Pim respectively. The ELISA assay (Figure 4A) and the dot blot results (Figure 4B) showed that Pim reduced secreted HBsAg. Similarly, the inhibition of intracellular HBsAg levels by Pim was also verified by western blot (Figure 4C). In agreement with the previous results of real-time PCR, Pim decreased the total HBV RNAs and HBV 3.5-kb RNA levels in a dose-dependent manner, whereas ETV exhibited almost no inhibitory effect (Figure 4D,E). Furthermore, Pim markedly decreased the levels of 3.5-kb, 2.4-kb and 2.1-kb HBV RNAs as evidenced by Northern blotting analysis (Figure 4F). Additionally, it turned out that a significant reduction of HBV core DNA was observed in HepG2-NTCP cells treated with Pim, possibly resulting from the decline of up-stream 3.5-kb RNA (Figure 4G,H). Of note, following HBV infection and episomal cccDNA formation, all HBV RNAs transcript from cccDNA in HepG2-NTCP. The HBV cccDNA level after Pim treatment was thus examined by Southern blot. The siPCNA was used as a positive control, showing a decrease in cccDNA level. The interference efficiency of PCNA was verified by western blot (Feng et al., 2019; Wei and Ploss, 2021) (Supplementary Figure S3B). The results showed that Pim had no obvious effect on the level of HBV cccDNA, compared to those treated with DMSO and ETV (Figure 4I and Supplementary Figure S3C).

### Pim Regulated cccDNA Transcription Related to Transcription Factors

**FIGURE 5:**
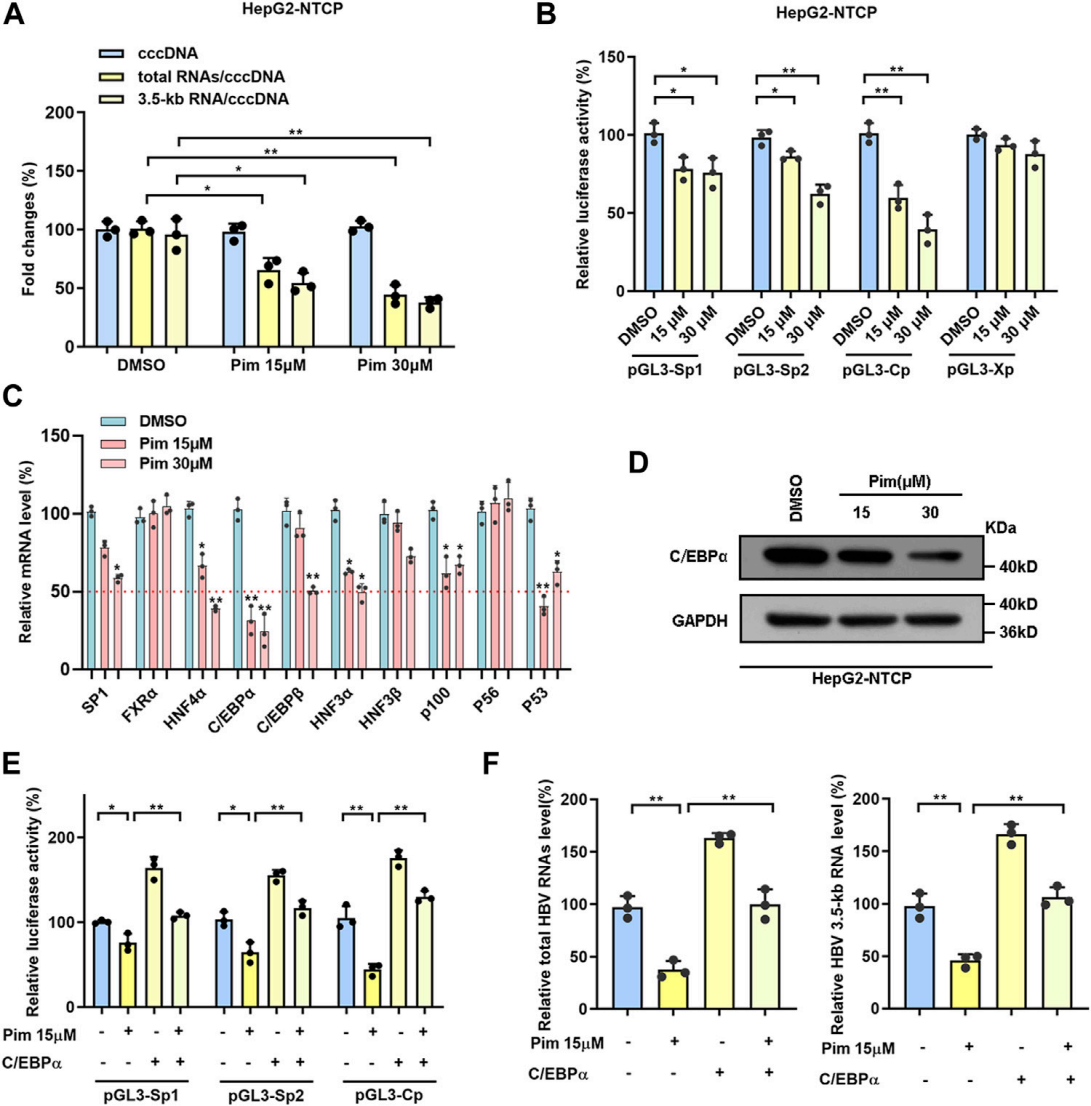
Pim regulated cccDNA transcription related to transcription factors **(A)** The ratios of total HBV RNAs/cccDNA and HBV 3.5-kb RNA/cccDNA were reduced in a dose-dependent manner **(B)** The activities of four HBV promoters were detected by dual-luciferase reporter assay system after Pim treated 48 h in HepG2-NTCP cells **(C)** The mRNA level of transcription factors related to HBV SpⅠ, SpⅡ and core promoters were detected in HepG2-NTCP by RT-PCR after Pim treatment **(D)** The change of protein level of C/EBPα was identified by Western blot in HepG2-NTCP **(E)** The luciferase report plasmids (pGL3-SpⅠ, pGL3-SpⅡ and pGL3-Cp) were transfected into HepG2-NTCP cells, accompanied by C/EBPα overexpression plasmid or not. 12 h after transfected, the cells were treated with 15 μM Pim as indicated for 48 h. The activities of HBV promoters were detected **(F)** The levels of total HBV RNAs and HBV 3.5-kb RNA were detected after Pim treating or C/EBPα overexpressing by qPCR (*p < 0.05; **p < 0.01).

Accumulating evidence has confirmed that Pim treatment significantly reduced the ratios of total HBV RNAs/cccDNA and HBV 3.5-kb RNA/cccDNA without affecting the level of cccDNA (Figure 4I and Figure 5A), indicating the anti-HBV effect of Pim in relation to transcriptional inhibition. To further explore the mechanism underlying the HBV markers reduction seen with Pim treatment, we examined the effect of Pim on four HBV promoters that closely associated with cccDNA transcription. Consistently, the data showed that Pim significantly reduced SpI, SpII and core promoter activities. It is well-known that transcription factors function by interacting with DNA sequences upstream of the gene to control transcriptional activity, where regulation can be achieved by initiation or inhibition of transcription, thus the mRNA levels of a group of transcription factors associated with HBV SpI, SpII and core promoter in HepG2-NTCP cells were monitored (Figure 5B) (Quasdorff and Protzer, 2010; Turton et al., 2020). When subjected to Pim treatment, the mRNA expression of C/EBPα was declined significantly in comparison with other transcriptional factors (Figure 5C). Subsequently, the expression of C/EBPα was further assessed by western blot (Figure 5D), showing that reduction of C/EBPα occurred in both transcription and translation levels. Previous studies have reported C/EBPα as an important transcriptional factor of HBV cccDNA and as a controller of HBV precore/core promoter, HBV enhancer II and S promoter (Bock et al., 1999; Choi et al., 1999; Turton et al., 2020). To investigate the role of C/EBPα in the HBV transcription-regulated by Pim, we overexpressed C/EBPα in HepG2-NTCP cells, and the overexpression efficiency of C/EBPα was verified by western blot (Supplementary Figure S4A,B). The results revealed that the inhibitory effect of Pim on HBV promoters could be reversed by C/EBPα overexpression (Figure 5E). Moreover, we found that overexpression of C/EBPα also significantly reversed the level of HBV RNAs which was reduced by Pim treatment (Figure 5F). Consequently, these data revealed that the anti-HBV effect of Pim was related to transcription factors, mainly involving the transcription factor C/EBPα.

### Anti-HBV Activity of Pim *in vivo*

**FIGURE 6:**
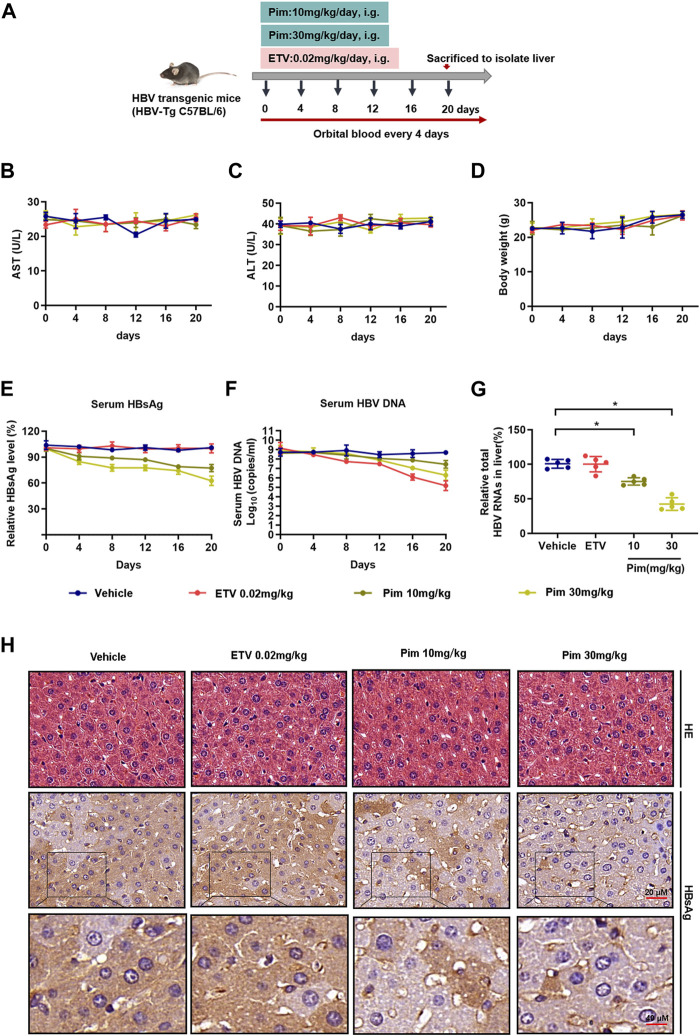
Anti-HBV activity of Pim *in vivo* **(A)** Flow chart explaining the way and concentration of Pim and ETV administration as well as the intervals of orbital blood collection, in HBV transgenic mouse model **(B–C)** The level of ALT and AST were detected by colorimetric microplate assay **(D)** The bodyweight with Pim administration was monitored **(E)** Serum HBsAg was quantified by ELISA assay **(F)** Serum HBV DNA was detected by quantification PCR **(G)** Total HBV RNAs in the liver tissue were determined by real-time PCR **(H)** Representative images of immunohistochemistry of HBsAg in liver tissue. HE staining of all groups as a control (*p < 0.05; **p < 0.01).

As demonstrated in *in vitro* studies, Pim down-regulated cccDNA transcription and inhibited the production of HBsAg. Based on its anti-HBV potential in HBV stably expressing cells and HBV infection cells, we further investigated the anti-HBV effect of Pim *in vivo*. HBV transgenic mice were applied in the study, and the dosage and method of oral administration of Pim were chosen based on the previous reports and preliminary experiment (Du et al., 2007; Guzman et al., 2014; Nonaka et al., 2015) (Supplementary Figure S5A). It turned out that Pim with a dose of 10 mg/kg and above showed a reduction of HBV markers. At the same time, Pim with a dose up to 30 mg/kg showed no significant toxicity. The schematic depiction of the animal experiment is shown in Figure 6A. Before assessing the antiviral effect of Pim in the mouse model, the oral toxicity of Pim on mice was tested, by the liver function index serum ALT and AST, showing a fluctuation within the normal range (Figure 6B,C). Besides, no significant change of body weight after Pim administration was found (Figure 6D). This demonstrated that no obvious hepatotoxicity was found upon treatment with Pim at either concentration.

The antiviral effect of Pim against HBV infection was subsequently assessed *in vivo*. As shown in Figure 6E, Pim-treated groups markedly decreased circulating HBsAg levels compared to the control. Moreover, ETV treatment reduced the serum level of HBV core DNA as expected. The levels of serum HBV core DNA were reduced by 12.89 and 24.74% respectively in the groups treated with 10 mg/kg Pim and 30 mg/kg Pim (Figure 6F). It’s worth noting that Pim decreased intrahepatic total HBV RNAs and HBV 3.5 kb RNA after complete treatment, and this observation was in line with our *in vitro* RNA analysis (Figure 6G and Supplementary Figure S5B). Finally, the results of HE staining showed that there were no obvious pathological changes in the liver sections of all groups. Immunohistochemistry was also conducted to observe the HBsAg in liver tissue (Figure 6H). Consistent with the previous results, Pim treatment significantly diminished the intrahepatic HBsAg level compared to the vehicle or ETV treatment groups. Taken together, Pim exhibited anti-HBV activity without hepatotoxicity.

## Discussion

Currently, IFNs and NAs are the recommended first-line drugs for suppressing HBV viral replication in chronically infected patients. However, the ideal goal of “functional cure” is rarely achieved (Kang et al., 2019), and thus the discovery of compounds targeting different stages of the HBV life cycle is still under research. As an irreplaceable biomarker of “functional cure”, developing new agents for inhibiting HBsAg has been reported. For example, RG7834, which is a novel orally available inhibitor of HBsAg with high selective for HBV, suppressed the HBsAg level without affecting the gene expression levels and the functions of transcription factors in infected hepatocytes (Mueller et al., 2018). However, the target and the specific mechanism of RG7834 remained to be investigated. REP-2139 and REP-2165, a kind of nucleic acid polymers under clinical trials, were also reported to be HBsAg secretion inhibitors. However, reduction in platelet counts, as well as ALT and AST flares was frequently reported (33%–68%), and the mechanism of action was not well understood (Fanning et al., 2019; Smolders et al., 2020). Notably, the high HBsAg levels were well-known to contribute to HBV-specific T-cell exhaustion and the direct negative regulation of immune cells, which exhibited the potential role in regulating anti-viral innate immune response in CHB. Thus, the initial goal of any future therapy for chronic HBV should involve the suppressions of intracellular and circulating HBsAg levels to restore patients’ antiviral immunity (Mueller et al., 2018).

To discover compounds potentially inhibiting HBsAg, a screening of an FDA-approved compound library was conducted, and Pim was identified as the most potent inhibitor with relatively low toxicity. Typically, more than 90% of drug candidates failed during research and development (Hay et al., 2014; Sekiba et al., 2019). Repositioning of FDA-approved drugs, which have already passed toxicity and other tests for clinical application, may enhance the success rate and reduce the costs associated with *de novo* drug development*.* Therefore, compared with other new anti-HBV drugs being developed, Pim may be applied to HBV-infected patients without high cost and time-consuming basic tests (Sekiba et al., 2019), allowing Pim to be an attractive and readily available anti-HBV candidate.

Of note, Pim was originally approved as an orally administered drug for the treatment of congestive heart failure. Recently, Pim has been reported to exhibit an antiplatelet effect *in vitro*, while it appears not to confer a risk for bleeding (Shipley et al., 2013). One study demonstrated that Pim enhanced glucose-induced insulin release, via calcium sensitization, in a dose-dependent manner (Fujimoto et al., 1998). Additionally, Pim was the most potent inhibitor of nitric oxide accumulation (Matsumori et al., 1996). It is worth noting that Pim also has beneficial effects on immune system by inhibiting the activity of transcription factor NF-κB and reducing cytokine production, such as TNF-α, IL-1β, IL-6 and nitric oxide (Iwasaki et al., 1999; Matsumori et al., 2000). The innate immune system is the first line of host defense against HBV infection, and hence plays a vital role in anti-HBV. The present consensus is that the viral clearance and disease pathogenesis during HBV infection are associated with the appearance of a vigorous T cell response to all viral proteins (Wieland et al., 2004). Future study on the regulation of T-cell immune responses by Pim for HBsAg clearance would further assess its applicability in chronic HBV treatment.

According to the preliminary results, Pim was screened out as a potential anti-HBV agent due to inhibition of supernatant HBsAg level. Further experiments confirmed that Pim could suppress total HBV RNAs, HBV 3.5-kb RNA, HBsAg and HBV core DNA in HBV stably expressing cell line HepG2.2.15 and HBV infection cell model HepG2-NTCP. In addition, a significant anti-HBV activity of Pim was also found in the HBV-transgenic mouse model. Mechanistically, Pim exhibited the activity of decreasing the level of HBV RNAs without affecting the level of cccDNA. This suggests that Pim targets cccDNA transcription suppression for antiviral effects. Although cccDNA elimination is the most efficient and direct strategy, it is unlikely to be achieved pharmacologically, and therefore transcriptional repression of cccDNA is considered as an alternative approach (Cheng et al., 2021). Accordingly, transcription factors are the primary determinants of transcription regulation. We found that C/EBPα, a known HBV-associated transcription factor, decreased most apparently after Pim treatment. Moreover, the inhibitory effect of Pim on HBV promoters could be reversed by overexpression of C/EBPα, suggesting that the anti-HBV effect of Pim was related to C/EBPα-mediated transcriptional regulation. However, other mechanisms responsible for the effects of Pim on HBV transcription and replication should be considered. Pim also moderately decreased the mRNA levels of other transcription factors related to cccDNA transcription, such as SP1, HNF4α, and HNF3α. According to previous reports, Sp1 was a ubiquitous transcription factor. HNF3α was a liver-related transcription factor, and HNF4α was a nuclear receptor in hepatocytes. All of the above-mentioned transcription factors could up-regulate the transcription activity of the related HBV promoters. Therefore, we proposed that the inhibitory effect of Pim on cccDNA transcription was not solely relied on C/EBPα, and the in-depth molecular mechanism was yet to be elucidated.

Since transcription factors are generally involved in the regulations of other gene expression, the possible side effects of Pim should also be taken into consideration. Further studies would be needed to investigate the alternations of host-wide gene expression levels upon Pim treatment. In addition, Pim is an FDA approved drug with no apparent toxicity observed in our experiments, but the drug safety for long-term usage remains to be determined. In this study, the effects of Pim against HBV observed were modest, but its effect was also significant. Further studies on docking *in silico* and chemical modification may improve its antiviral effects.

To our knowledge, the inhibitory effect of Pim on HBV transcription and translation was first reported *in vitro* and *in vivo*, along with the subsequent investigations of the underlying mechanism, it is, the anti-HBV effect of Pim in relation to transcription factors, of which the transcription factor C/EBPα is mostly implicated. Therefore, Pim may provide a promising novel drug against HBV infection.

## Abbreviations

ALT, alanine transaminase; AST, aspartate transaminase; CHB, Chronic hepatitis B; HBV, hepatitis B virus; CC50, half maximal cytotoxicity concentration; C/EBPα, CAAT enhancer-binding protein α; cccDNA, covalently closed circular DNA; DIG, digoxin; DMSO, dimethyl sulfoxide; ELISA, enzyme-linked immunosorbent assay; HBsAg, hepatitis B surface antigen; HNF, hepatocyte nuclear factor; EC50, concentration for 50% of maximal effect; ETV, entecavir; PHH, primary human hepatocytes; NAs, nucleoside analogs; GAPDH, glyceraldehyde-3-phosphate dehydrogenase; IFNs, interferons; i.g., intragastric administration; Pim, pimobendan; PBS, phosphate buffered saline; MTT, 3-(4,5-Dimethylthiazol-2-yl)-2,5-diphenyltetrazolium bromide; NTCP, Na+-taurocholate cotransporting polypeptide; SP1, specificity protein 1; SpI, preS1 promoter; rcDNA, relaxed-circular partially double-stranded DNA; SpII, preS2 promoter; PCNA, proliferating cell nuclear antigen.

## Data Availability

The original contributions presented in the study are included in the article/Supplementary Material, further inquiries can be directed to the corresponding authors.

## Abbreviations

ALT, alanine transaminase; AST, aspartate transaminase; CHB, Chronic hepatitis B; HBV, hepatitis B virus; CC50, half maximal cytotoxicity concentration; C/EBPα, CAAT enhancer-binding protein α,; cccDNA, covalently closed circular DNA; DIG, digoxin; DMSO, dimethyl sulfoxide; ELISA, enzyme-linked immunosorbent assay; HBsAg, hepatitis B surface antigen; HNF, hepatocyte nuclear factor; EC50, concentration for 50% of maximal effect; ETV, entecavir; PHH, primary human hepatocytes; NAs, nucleoside analogs; GAPDH, glyceraldehyde-3-phosphate dehydrogenase; IFNs, interferons; i.g., intragastric administration; Pim, pimobendan; PBS, phosphate buffered saline; MTT, 3-(4,5-Dimethylthiazol-2-yl)-2,5-diphenyltetrazolium bromide; NTCP, Na±taurocholate cotransporting polypeptide; SP1, specificity protein 1; SpI, preS1 promoter; rcDNA, relaxed-circular partially double-stranded DNA; SpII, preS2 promoter; PCNA, proliferating cell nuclear antigen.
