# Supplementary figures and images for: Pimobendan Inhibits HBV Transcription and Replication by Suppressing HBV Promoters Activity
Source: Front Pharmacol. 2022 Jun 3;13:837115. doi: 10.3389/fphar.2022.837115 (PMC9204083; doi:10.3389/fphar.2022.837115)

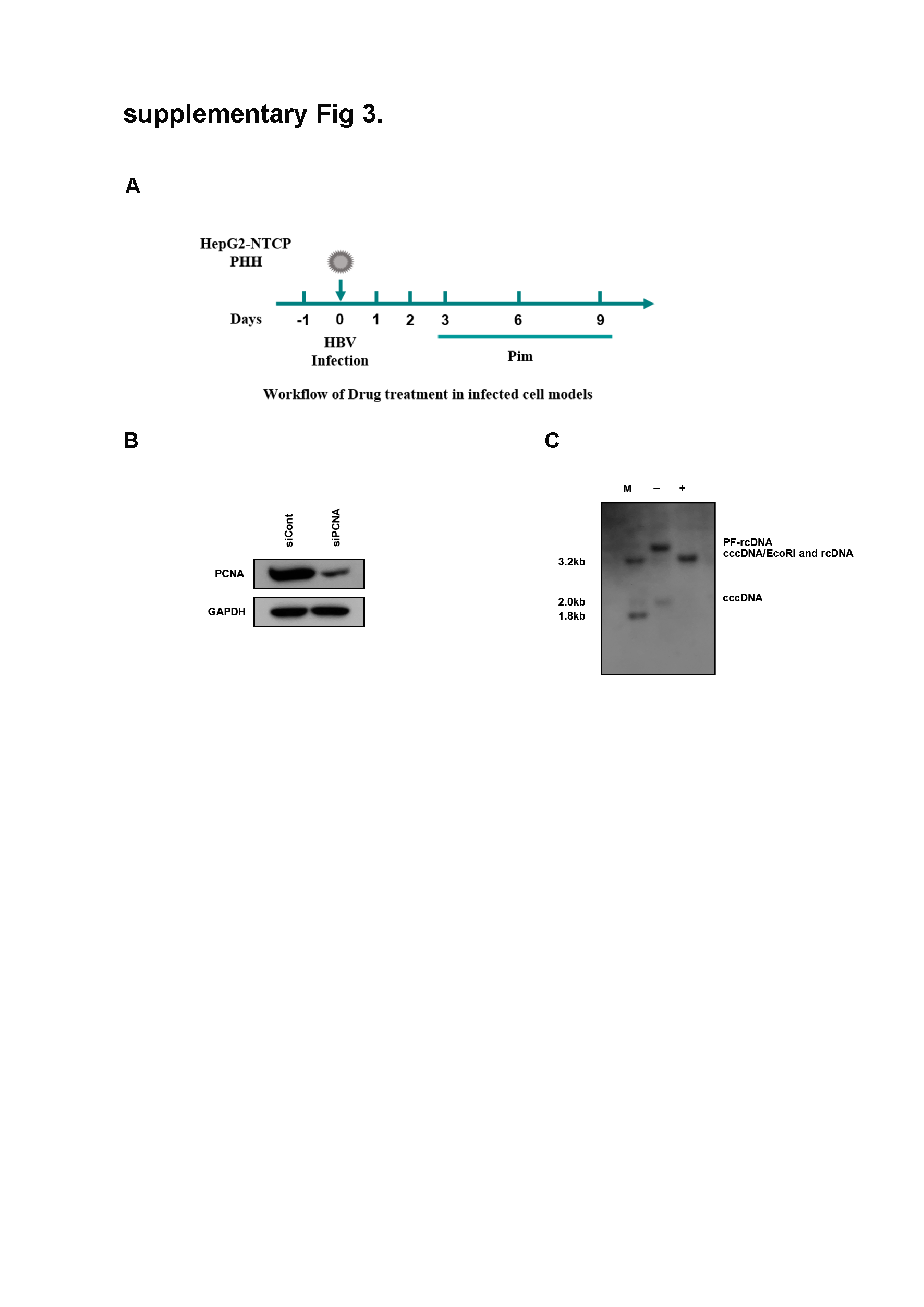

Supplement: Supplementary file 1 [file Image3.TIF]

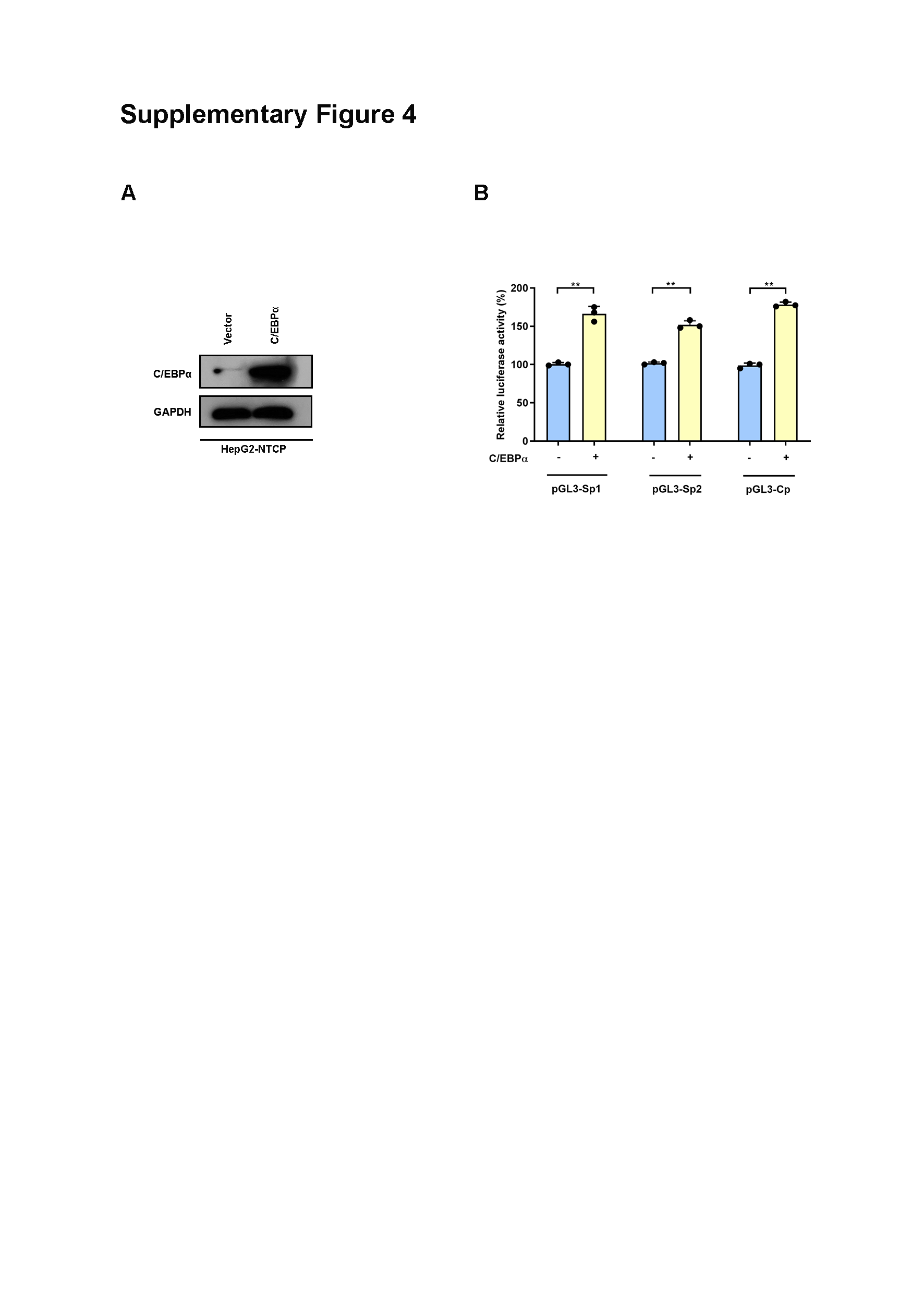

Supplement: Supplementary file 2 [file Image4.TIF]

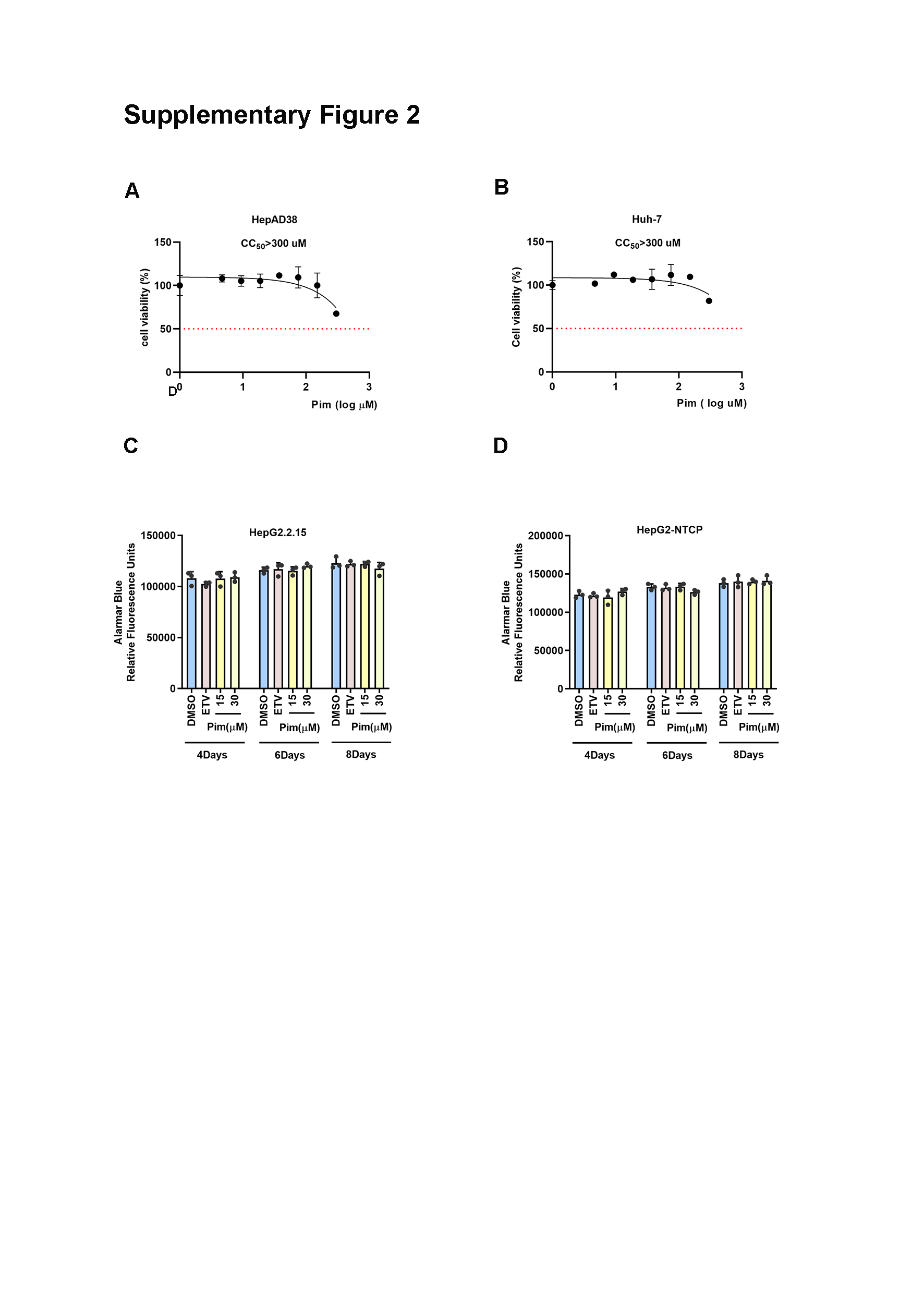

Supplement: Supplementary file 3 [file Image2.TIF]

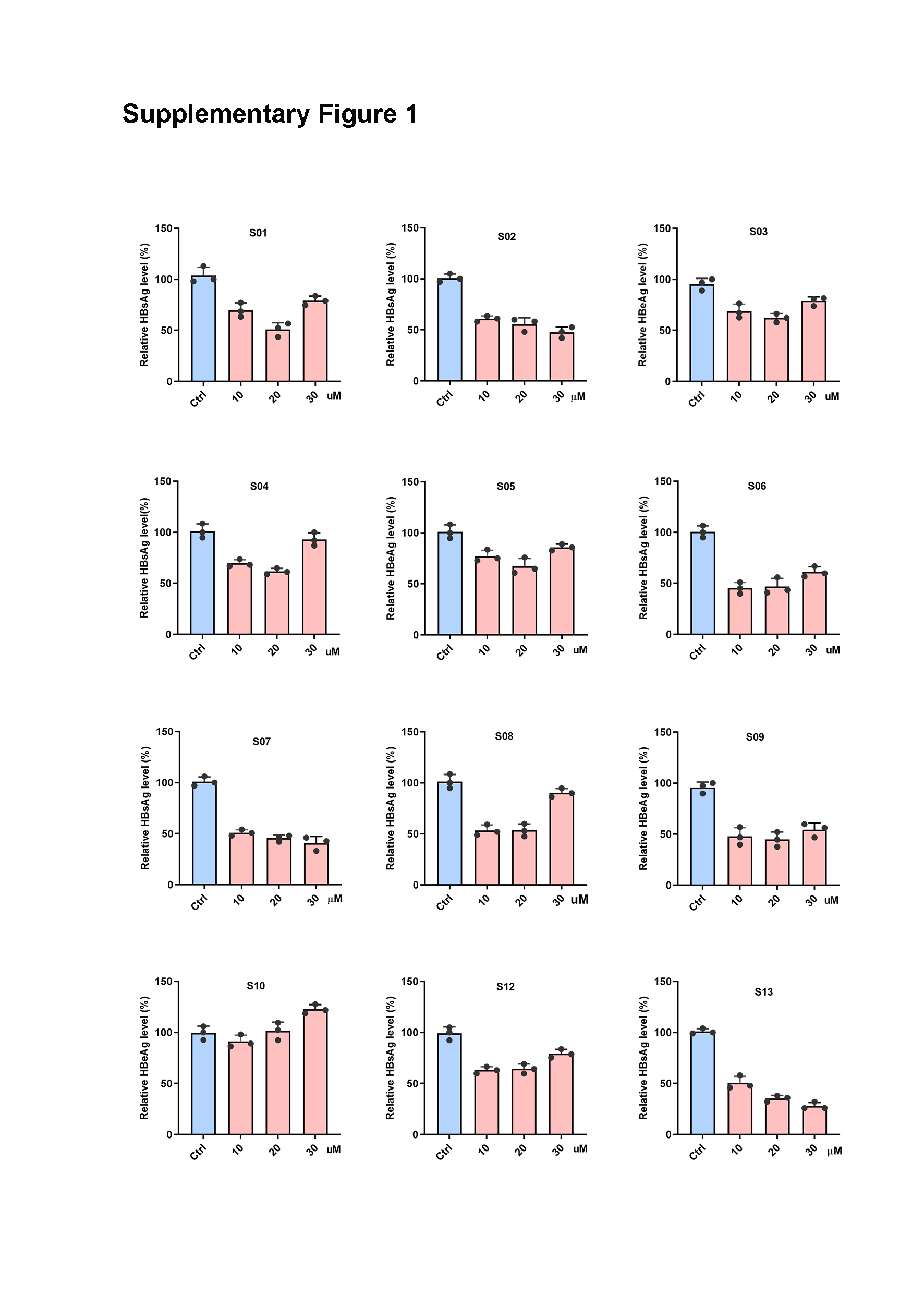

Supplement: Supplementary file 4 [file Image1.TIF]

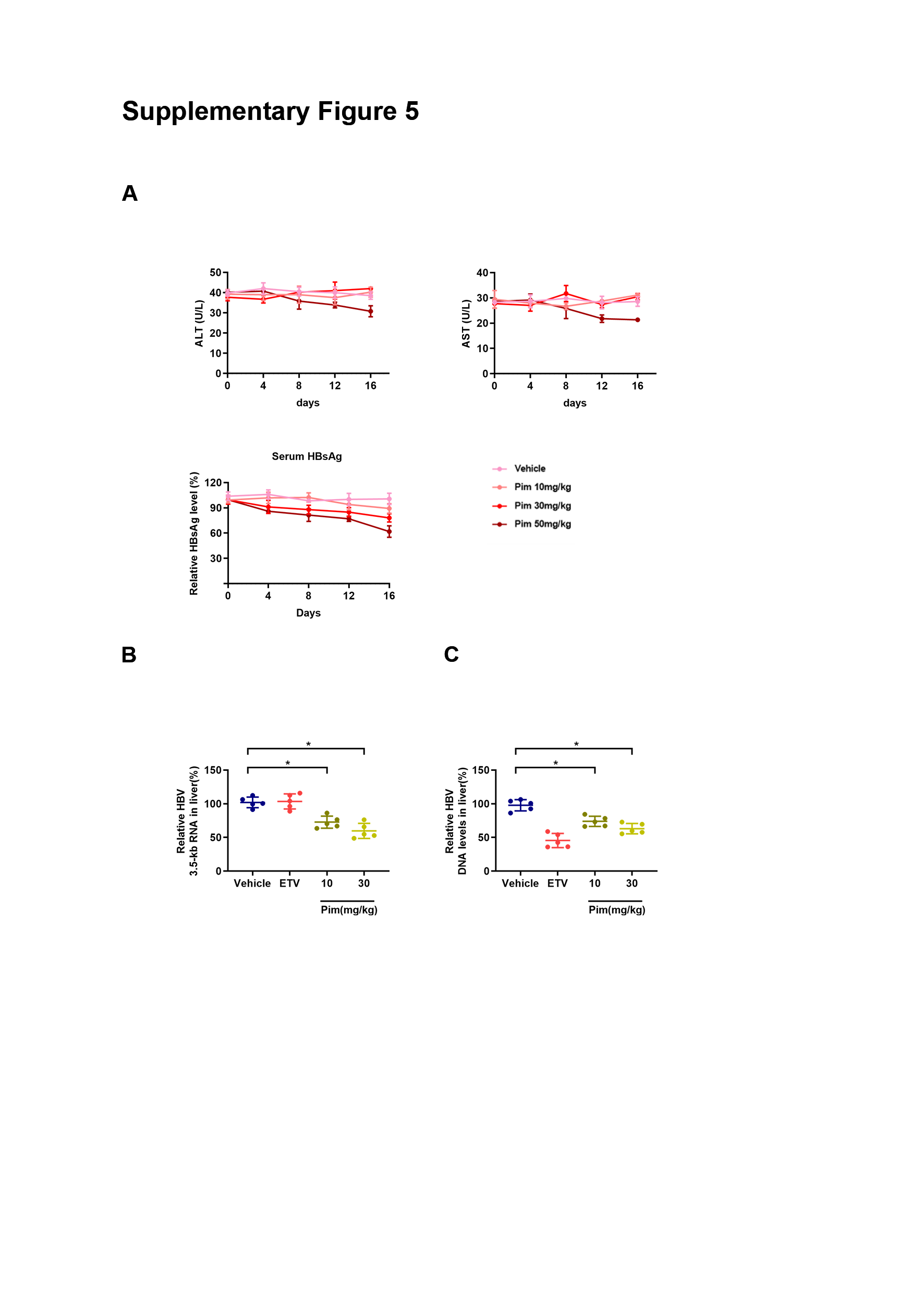

Supplement: Supplementary file 5 [file Image5.TIF]
