# Supplementary material for: Pimobendan Inhibits HBV Transcription and Replication by Suppressing HBV Promoters Activity
Source: Front Pharmacol. 2022 Jun 3;13:837115. doi: 10.3389/fphar.2022.837115 (PMC9204083; doi:10.3389/fphar.2022.837115)
Supplement: Supplementary file 6 [file DataSheet1.docx]

Supporting Information

**Fig. S1**

**Fig.S1. Four compounds inhibited HBsAg production in a dose-dependent manner.** The relative HBsAg levels were detected by ELISA assay. Compounds of 10, 20 or 30 μM were treated to HepG2.2.15, including Pim, only four compounds inhibited HBsAg in a dose-dependent manner.

**Fig. S2**

**Supplementary Fig.2** **Cytotoxicity of Pim in different cell lines.** The cytotoxicity was determined by the MTT assay in HepAD38 cells (A) Huh-7 cells (B). (C-D) Cytotoxicity was further detected in HepG2.2.15 cells at day 4, 6 and 8 (HepG2-NTCP cells at day 4, 6, 8) by Alamar Blue assay. Experiments were independently repeated at least three times, and representative data was exhibited as the means ± SD.

**Fig. S3**

**Supplementary Fig.3. Anti-HBV effects of Pim in HBV infection cell model.** (A) Flowchart of Drug treatment in infected cell models. (B) The Western bot assay was used to verify the interference efficiency of siPCNA. (C) the EcoRI digested-Hirt DNA and EcoRI undigested-Hirt DNA were examined by Southern blot analysis.

**Fig. S4**

**Supplementary Fig.4. Overexpressed C/EBPα increased the activity of three HBV promoters.** (A) Transfection of overexpression C/EBPα plasmid into HepG2-NTCP cells, and the overexpression efficiency was verified by Western blot. (B) The luciferase report plasmids (pGL3-SpⅠ, pGL3-SpⅡ and pGL3-Cp) were transfected into HepG2-NTCP cells, accompanied by transfection of C/EBPα overexpression plasmid, 48h after transfected, the activities of HBV promoters were detected.

**Fig. S5**

**Supplementary Fig.5 The effect of Pim in vivo.** (A) The preliminary experiment in HBV transgenic mice. HBV transgenic mice were treated with PBS, 10, 30 or 50 mg/kg Pim respectively. The level of ALT and AST were detected by colorimetric microplate assay, and Serum HBsAg was quantified by ELISA assay. (B) HBV 3.5-kb RNA in the liver tissue was determined by real-time PCR. (C) HBV core DNA in the liver tissue was observed by the quantitative PCR

Supplementary Table 1

| No. | $\boldsymbol{CC}_{\boldsymbol{50}}$(mM) | HBsAg secretion (%) | No. | $\boldsymbol{CC}_{\boldsymbol{50}}$(mM) | HBsAg secretion (%) |
| --- | --- | --- | --- | --- | --- |
| S01 | 87.2 | 35.10 | S08 | >100 | 33.41 |
| S02 | >100 | 44.57 | S09 | 82.4 | 35.38 |
| S03 | 100 | 36.83 | S10 | >100 | 48.02 |
| S04 | >100 | 30.62 | S11 | >100 | 45.86 |
| S05 | 93.6 | 41.51 | S12 | >100 | 44.23 |
| S06 | >100 | 41.91 | S13 | 93 | 31.82 |
| S07 | >100 | 47.90 |  |  |  |

Data are expressed as means ± standard deviations.

Supplementary Table 2

| HBV core DNA forward | 5’-CCTAGTAGTCAGTTATGTCAAC-3’ |
| --- | --- |
| HBV core DNA reverse | 5’-TCTATAAGCTGGAGGAGTGCGA-3’ |
| transgenic mouse serum and liver DNA forward | 5’-CCTCTTCATCCTGCTGCT-3 |
| transgenic mouse serum and liver DNA reverse | 5’-AACTGAAAGCCAAACAGTG-3’ |
| total HBV RNAs forward | 5’- ACCGACCTTGAGGCATACTT-3’ |
| total HBV RNAs reverse | 5’- GCCTACAGCCTCCTAGTACA-3’ |
| HBV 3.5-kb mRNA forward | 5’- GCCTTAGAGTCTCCTGAGCA-3’ |
| HBV 3.5-kb mRNA reverse | 5’- GAGGGAGTTCTTCTTCTAGG-3’ |
| β-actin forward | 5’-CTCTTCCAGCCTTCCTTCCT-3’ |
| β-actin reverse | 5’- AGCACTGTGTTGGCGTACAG-3’ |
| β-actin mRNA（mouse）forward | 5’- CCACCATGTACCCAGGCATT-3’ |
| β-actin mRNA（mouse）reverse | 5’-CGGACTCATCGTACTCCTGC-3 |
| cccDNA forward | 5’- CTCCCCGTCTGTGCCTTCT-3’ |
| cccDNA reverse | 5’-CCCCAAAGCCACCCAAG-3’ |
| cccDNA probe | 5’-TTCATCCTGCTGCTATGCCTGATCTTCTTG-3’ |
| SP1 forward | 5’-CTGAAGCTGGGTAGCCTATTG-3’ |
| SP1 reverse | 5’-CTACTGCTGCGACCTTTCTT-3’ |
| FXRα forward | 5’-GGACATGCAGATGGACAAGA-3’ |
| FXRα reverse | 5’-CCCTTGGAGTCAGGGTTAAA-3’ |
| HNF4 forward | 5’-GCCTACCTCAAAGCCATCAT-3’ |
| HNF4 reverse | 5’-CGGTCGTTGATGTAGTCCTC-3’ |
| C/EBPα forward | 5’-CGAGCCAGGACTAGGAGATT-3’ |
| C/EBPα reverse | 5’-CCTCATCTTAGACGCACCAA-3’ |
| C/EBPβ forward | 5’-CTGGAGACGCAGCACAAG-3’ |
| C/EBPβ reverse | 5’-ACAGCTGCTCCACCTTCTTC-3’ |
| HNF3α forward | 5’-CAGCAAACAAAACCACACAAACC-3’ |
| HNF3α reverse | 5’-ACACTTGTGGATCATTAAACTTCGC-3’ |
| HNF3β forward | 5’-GTTGTTGTTGTTCTCCTCCATTGC-3’ |
| HNF-3β reverse | 5’-AACTACATGGTTTTACACCGAGTCAC-3’ |
| NF-κB (p100) forward | 5’-GTCCAGGGTATAGCTTCCCA-3’ |
| NF-κB (p100) reverse | 5’-CACAACCTTCAGGGTCCTTT-3’ |
| p56 forward | 5’-CCTGTCCTTTCTCATCCCAT-3’ |
| p56 reverse | 5’-CCTCTTTCTGCACCTTGTCA-3’ |
| p53 forward | 5’-AACAACACCAGCTCCTCTCC-3’ |
| p53 reverse | 5’-CTCATTCAGCTCTCGGAACA-3’ |
|  |  |
| siRNA | Target Sequences (5’→ 3’) |
| PCNA-siRNA | 5’-GATGCTGTTGTAATTTCCTGT-3’ |
